# Molecular and pathological characterization of the *EZH2* rs3757441 single nucleotide polymorphism in colorectal cancer

**DOI:** 10.1186/s12885-015-1889-2

**Published:** 2015-11-09

**Authors:** Lorenzo Fornaro, Pinuccia Faviana, Veronica De Gregorio, Caterina Vivaldi, Elisa Paolicchi, Gianluca Masi, Fotios Loupakis, Elisa Sensi, Cristiana Lupi, Gabriella Fontanini, Yuzhuo Wang, Romano Danesi, Alfredo Falcone, Francesco Crea

**Affiliations:** 1 Unit of Medical Oncology 2, Azienda Ospedaliero-Universitaria Pisana, Istituto Toscano Tumori, Pisa, Italy; 2 Unit of Pathology, Department of Surgical, Medical and Molecular Pathology and Critical Care, University of Pisa, Pisa, Italy; 3 Department of Biology, Unit of Genetics, University of Pisa, Pisa, Italy; 4 Experimental Therapeutics, BCCA Cancer Research Centre, Vancouver, BC Canada; 5 Vancouver Prostate Centre, Vancouver, BC Canada; 6 Pharmacology Unit, Department of Clinical and Experimental Medicine, University of Pisa, Pisa, Italy; 7 Department of Life Health and Chemical Sciences, The Open Universit, Milton Keynes, UK

**Keywords:** *BRAF*, Colorectal cancer, *EZH2*, *KRAS*, Polycomb, Single nucleotide polymorphism

## Abstract

**Background:**

The enhancer of zeste-homolog 2 (*EZH2*) is involved in cancer development through gene silencing by trimethylation of lysine 27 of histone 3 (H3K27me3). The C/C genotype for the *EZH2* rs3757441 single-nucleotide polymorphism (SNP) is linked with poor prognosis in metastatic colorectal cancer (CRC), but molecular and pathological characterization of this SNP is lacking.

**Methods:**

119 primary CRCs were analyzed. SNP was evaluated by real-time PCR from colonic healthy tissue, while EZH2 and H3K27me3 expression were studied by immunohistochemistry. We primarily looked for correlation between *EZH2* rs3757441 genotypes and EZH2/H3K27me3 expression. Potential associations between EZH2/H3K27me3 expression and clinico-pathological features or *KRAS* exon 2 and *BRAF* exon 15 mutations were secondary endpoints. Statistical analysis was performed by chi-square test, T-test or ANOVA.

**Results:**

The C/C genotype was significantly associated with higher EZH2 (100 *vs.* 44 %; *P* = 0.019) and H3K27me3 (100 *vs.* 38 %; *P* = 0.009) staining intensity compared with C/T and T/T. EZH2 3+ staining significantly correlated with stronger H3K27me3 expression (*P* = 0.039). *KRAS* and *BRAF* mutations were not associated with EZH2 or H3K27me3 expression.

**Conclusion:**

*EZH2* rs3757441 C/C genotype is associated with stronger EZH2 and H3K27me3 immunoreactivity in primary CRC: this SNP may serve as a promising biomarker for EZH2-targeting agents and may add independent information to *KRAS* and *BRAF* testing.

**Electronic supplementary material:**

The online version of this article (doi:10.1186/s12885-015-1889-2) contains supplementary material, which is available to authorized users.

## Background

Colorectal cancer (CRC) is one of the leading causes of cancer-related deaths worldwide [1]. Validated predictive or prognostic biomarkers in metastatic CRC (mCRC) are limited to *RAS* and *BRAF* mutations [2, 3]. There is therefore a dire need for a deeper characterization of CRC biology and for the identification of innovative therapeutic targets.

Epigenetics offers a different perspective to understand cancer biology, complementing the conventional genetic approach [4]. Polycomb Group genes (PcGs) represent some of the most studied components of the epigenetic machinery. PcGs are organized in two major Repressive Complexes (PRCs) and induce gene silencing through histone post-translational modifications [5]. Most PcGs have been involved in the initiation and promotion of cancer and contribute to drug resistance in both haematological and solid malignancies [6].

EZH2 is the catalytic subunit of PRC2, which catalyzes histone H3 trimethylation on lysine 27 (H3K27me3), thereby promoting selective gene silencing in normal stem cells and cancer cells. This epigenetic modification is responsible for several key events in tumor development, including the occurrence of distant metastases and angiogenesis [6, 7]. We have recently investigated the role of *EZH2* single-nucleotide polymorphisms (SNPs) in mCRC patients treated with chemotherapy plus bevacizumab: the C/C genotype for the rs3757441 SNP was associated with worse prognosis in terms of progression-free and overall survival [8]. This finding was subsequently confirmed in patients treated with chemotherapy alone, an evidence suggesting a prognostic significance of this SNP in mCRC [9]. We hypothesized that the C/C genotype would create a transcription factor-binding site, thereby increasing the expression of the oncogenic EZH2 protein [8]. However, a definitive characterization of rs3757441 is still missing, since correlation between the SNP variants and EZH2/H3K27me3 protein expression in CRC samples has not been reported.

In the present study, we analyzed *EZH2* rs3757441 genotype, EHZ2/H3K27me3 expression and pathological and molecular characteristics of 119 primary CRC samples.

## Methods

### Sample selection and DNA isolation

Consecutive CRC patients who underwent surgery on primary tumor and had their samples available for analyses at a single Institution (Unit of Pathology, Pisa) were identified. All patients provided written informed consent for sample collection and analysis. The study was conducted in accordance to the Declaration of Helsinki and the protocol was approved by the Ethics Committee of Pisa University Hospital. A total of 119 formalin-fixed paraffin-embedded tissues of primary CRCs were used for DNA extraction. DNA used for SNP analysis was extracted from normal colonic tissue, while tumor DNA was sequenced for *KRAS* and *BRAF* analyses. DNA was isolated using the QIAamp DNA Mini Kit (QIAGEN), following manufacturer’s instructions. Concentration and purity of DNA were measured through a spectrophotometer.

### Genetic analyses

Researchers who were not aware of EZH2 and H3K27 immunohistochemistry results and were blinded to patients’ clinical data performed genetic analyses. *EZH2* SNP [c.626–394C > T (rs3757441)] was analyzed through real-time PCR, as previously described [8]. *KRAS* exon 2 (codon 12–13) and *BRAF* exon 15 (codon 600) mutations were detected via real-time sequencing using PyroMark Gold Q96 reagents (QIAGEN) on PyroMarkTM Q96 ID instrument (Biotage, Sweden), as reported elsewhere [10]. Results were analyzed with PyroMark Q24 1.0.9 software.

### EZH2 and H3K27 immunohistochemistry

Immunohistochemistry was performed on formalin fixed, paraffin-embedded tumor tissues, using previously validated protocols for both antibodies [11]. Involved pathologist carefully reviewed all CRC samples and selected tumor sections which were most representative of each tumor. Briefly, 5 μm-thick tissue sections were deparaffinized in xylene and rehydrated in a graded ethanol series. Slides were stained using a diaminobenzidine detection system preceded, only for EZH2, by heat-induced epitope retrieval involving immersion of tissue sections in a pre-warmed buffer solution (Target Retrieval Solution, DakoCytomation, Carpinteria, CA) and maintaining heat in a steamer at 988 °C for 50 min. To reduce nonspecific staining caused by endogenous biotin, the Endogenous Biotin Blocking Kit (Ventana Medical Systems, SA, Illkirch, Cedex, France) was employed, according to Manufacturer’s instructions. EZH2 and H3K27me3 immunostaining was performed using monoclonal antibodies by Abcam (1:100 dilution and 1:50 dilution, respectively). Negative controls were obtained by omission of the primary antibodies.

Two pathologists were responsible for immunohistochemical evaluation and provided independent scoring results for both EZH2 and H3K27. In case of discrepancy, joint revision of the ambiguous samples was performed to assure the consistency of definitive results.

EZH2 expression was categorized as: *i*) percentage of positive cells (0-100 %, evaluated as a continuous variable): the percentage of positive cells for each sample was determined as the average value of positive cells in 10 fields (approximately 100 cells per field were counted) at 10 High Power Field (HPF); *ii*) staining intensity, evaluated by a validated system [12]: we classified all cases in 4 categories [(−) less than 10 % positive cells, any intensity; (1+) from 10-25 %, any intensity; (2+) from 25-75 %, any intensity; (3+) greater than 75 %, any intensity]; *iii*) Staining Index (SI), a semiquantitative method, evaluating both the heterogeneous distribution of positive cells and the differing intensity of the staining simultaneously: as described by Fluge et al. [13], SI is calculated as the product (0 to 9) of positive cells (0, 0 %; 1, < 10 %; 2, 10–50 %; and 3, > 50 %) and staining intensity (negative to 3+).

H3K27me3 expression was scored as follows: *i*) percentage of positive cells (0 - 100 %); *ii*) staining intensity (as described for EZH2). In both cases, the pathologist performing immunohistochemistry analyses was blinded from genetic data.

### Statistical analysis

EZH2 and H3K27 expression intensities were correlated with clinical and molecular features by means of chi-square test, T-test or ANOVA (as appropriate; significance was set at *P* < 0.05 for two-tailed tests).

Neither pathologists performing immunohistochemistry nor molecular biologists performing genetic evaluation were directly involved in statistical analyses, which were conducted by independent investigators.

## Results

### EZH2 and H3K27me3 expression

**Fig. 1:**
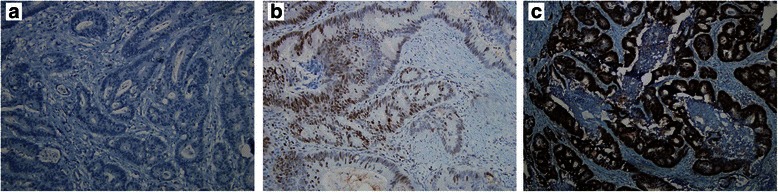
Different EZH2 staining intensity at immunohistochemistry. EZH2 negative (panel **a**), EZH2 2+ (panel **b**) and EZH2 3+ (panel **c**) cases are shown. Negative: no staining; 1+ (not shown): faint and barely perceptible staining; 2+: incomplete, weak to moderate staining; 3+: uniform, complete and intense staining

**Fig. 2:**
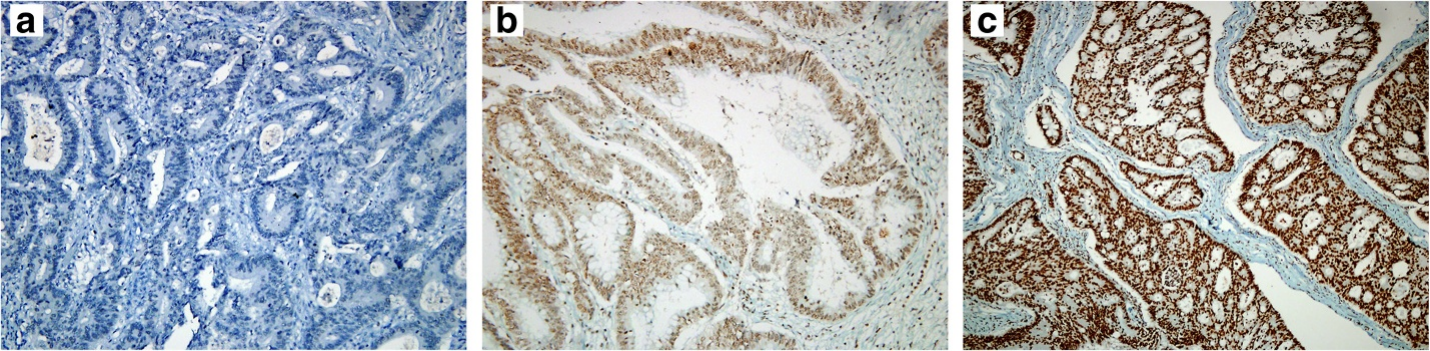
Different H3K27me3 staining intensity at immunohistochemistry. H3K27me3 negative (panel **a**), H3K27me3 2+ (panel **b**) and H3K27me3 3+ (panel **c**) are shown. Negative: no staining; 1+ (not shown): faint and barely perceptible staining; 2+: incomplete, weak to moderate staining; 3+: uniform, complete and intense staining

Results of the expression analysis (Figs. 1, 2) are summarized in Table 1. Prevalence of different SNP genotypes was in line with published data in Caucasian populations. Mean number of EZH2 or H3K27me3 positive cells was 46.2 % (standard deviation, SD: 28.99) and 64.2 % (SD: 29.10), respectively. *KRAS*-*BRAF* mutations occurred as expected from previous reports.

**Table 1:** Specimen characteristics (*n* = 119)

Feature	Number	Percent
Tumor stage		
I	5	4
II	41	34
III	52	44
IV	21	18
Tumor grade		
2	76	64
3–4	43	36
Tumor site		
right colon	52	44
left colon	45	38
rectum	22	18
Mucinous histology		
yes	37	31
no	82	69
*KRAS status*		
wild-type	67	56
mutant	52	44
*BRAF status*		
wild-type	108	91
mutant	11	9
*EZH2 rs3757441 genotype*		
C/C	5	4
C/T	51	43
T/T	63	53
*EZH2* staining intensity		
3+	55	46
2+	33	28
1+	14	12
negative	17	14
*H3K27me3* staining intensity		
3+	48	40
2+	40	34
1+	17	14
negative	14	12

*Abbreviations*: *n,* number; %, percentage

### Correlation of EZH2 rs3757441 variants with EZH2/H3K27me3 expression

Our primary goal was to investigate potential correlations between rs3757441 variants and EZH2/H3K27me3 expression. Moving from our previous reports [8, 9], we explored the association between protein expression and each genotype. Subsequently, we grouped the C/T and T/T genotypes (previously associated with better prognosis) and compared them with the C/C genotype (Table 2).

**Table 2:** Correlation of *EZH2* rs3757441 variants with EZH2/H3K27me3 expression

	EZH2						H3K27me3			
	3+ staining intensity		% positive cells		SI 4–9		3+ staining intensity		% positive cells	
Genotype (*n*)	*n* (%)	*P*	mean (SD)	*P*	*n* (%)	*P*	*n* (%)	*P*	mean (SD)	*P*
C/C (5)	5 (100%)		60.0 (15.8)		5 (100%)		5 (100%)		82.0% (4.472)	
C/T (51)	20 (39%)		46.0 (27.7)		38 (74%)		18 (35%)		60.0% (30.53)	
T/T (63)	30 (48%)	0.028	46.0 (30.3)	0.571	44 (70%)	0.328	25 (40%)	0.022	66.19% (28.54)	0.200
C/C (5)	5 (100%)		60.0% (15.8)		5 (100%)		5 (100%)		82.0% (4.472)	
C/T or T/T (114)	50 (44%)	0.019	46.0% (29.1)	0.288	82 (72%)	0.322	43 (38%)	0.009	63.42% (29.47)	0.163

*Abbreviation*s: %, percentage; *n,* number; *P, P*-value; *SD,* standard deviation; *SI*, Staining Index

We found significant differences among the three genotypes in terms of both EZH2 (3+ samples: 100 % *vs.* 39 % *vs.* 48 %; *P* = 0.028) and H3K27me3 (3+ samples: 100 % *vs.* 35 % *vs.* 40 %; *P* = 0.022) staining intensity. When the C/T and T/T cases were grouped, the samples harbouring the C/C genotype showed significantly stronger EZH2 positivity compared to other genotypes (3+ samples: 100 % *vs.* 44 %, respectively; *P* = 0.019). The same significant association was reported for H3K27me3 (3+ samples among C/C *vs.* C/T or T/T: 100 % *vs.* 38 %; *P* = 0.009). Moreover, we observed a trend toward higher percentage of EZH2 positive cells (60.0 % *vs.* 46.0 %; *P* = 0.288) and higher SI (SI 4–9: 100 % *vs.* 72%; *P* = 0.322) in C/C cases compared to C/T or T/T cases.

### Correlation of EZH2 rs3757441 variants with clinical and molecular characteristics

The C/C genotype was associated with higher tumor grade: all the 5 cases were scored as grade 3–4 tumors, while undifferentiated histology was reported in 37 % and 38 % of the C/T and T/T cases, respectively (*P* = 0.021) (Additional file 1: Table S1). We also observed a higher incidence of *BRAF* mutation among T/T cases (16 % *vs.* 0 % and 2 % among C/C and C/T, respectively; *P* = 0.030).

### Correlation of EZH2/H3K27me3 expression with clinical and molecular characteristics

We did not find any significant correlation between EZH2 expression and tumor stage, grading, histology and site of origin (Additional file 1: Table S2).

None of the investigated tumor features was associated with H3K27me3 expression, with the exception of tumor site (H3K27me3 3+ cases were indeed more frequent among left-sided cancers compared with right-sided or rectal cancers: 55 % *vs.* 33 % *vs.* 27 %; *P* = 0.028) (Additional file 1: Table S3). We also found a trend toward lower H3K27me3 expression in terms of percentage of positive cells (67.9 % *vs.* 57.7 %; *P* = 0.065) and staining intensity (3+ cases: 28 % *vs.* 47 %; *P* = 0.052) in poorly differentiated tumors.

*KRAS* and *BRAF* genotypes were not associated with EZH2 or H3K27me3 expression in our series.

Of note, we found a significant association between EZH2 and H3K27me3 staining intensity (*P* = 0.039). These results further corroborate the notion that higher EZH2 expression in CRC samples results in increased histone H3K27 methylation.

## Discussion

We have already reported about the association between the *EZH2* rs3757441 C/C genotype and worse prognosis in mCRC [8, 9] and showed that EZH2 mRNA expression (as measured in peripheral lymphocytes) was significantly higher among C/C patients [8]. To characterize the role of this SNP in neoplastic cells, we analyzed *EZH2* rs3757441 in normal colonic tissue (as the SNP accounts for germline variation in gene sequence) and evaluated EZH2 expression in tumor cells by immunohistochemistry in a series of 119 primary CRC. In this context, we found that the C/C genotype was associated with significantly higher EZH2 expression (Table 2). Potential mechanisms by which the rs3757441 SNP may affect EZH2 expression have been proposed elsewhere [8].

Intriguingly, we also observed a strong association between the C/C genotype and increased expression of H3K27me3, which is the functional readout of EZH2 methylating activity. This result indicates that carriers of the C/C genotype display higher EZH2 activity. In keeping with these findings, our data suggest that higher EZH2 expression results in increased EZH2 activation, as measured by H3K27me3 expression, which is higher in EZH2 3+ cases.

Among the investigated clinico-pathological features, we observed a higher percentage of H3K27me3 cases among left colon tumors, compared with right colon or rectal cancers. This association was not significant for EZH2, even though EZH2 3+ samples were more frequent among left-sided tumors. It is known that right- and left-sided CRCs differ from biological and clinical perspectives [14, 15]: however, the small sample size of our series suggests caution in deriving any conclusion.

Finally, we investigated if *KRAS*/*BRAF* mutations were associated with the EZH2 pathway, finding no significant correlation. Therefore, the suggested prognostic value of EZH2 expression and SNP appears to be independent of the *KRAS* and *BRAF* status: *EZH2* genotyping may therefore add additional information to the conventional molecular profile of CRC cases.

In the present series, we did not investigate the association between the *EZH2* SNP or the EZH2/H3K27me3 expression and survival outcome: patient heterogeneity in terms of disease stage, adjuvant treatments and length of follow up might have affected the results, leading to inconclusive or confounding findings.

Our study has several limitations that should be taken into account in the interpretation of the results. First, we retrieved only 5 patients with the C/C genotype for the *EZH2* rs3757441 SNP: therefore, our findings cannot be considered definitive and further analyses should be conducted in larger patient cohorts to confirm the association between *EZH2* SNP variants and protein expression. Notably, the results described in the present manuscript are in line with our previous report about mRNA analysis on peripheral lymphocytes from CRC patients [8]. This investigation revealed that EZH2 mRNA expression is higher in homozygous C allele carriers, with no difference between C/T and T/T subjects [8]. In our opinion, these two investigations support further evaluation of this SNP in CRC and possibly in other malignancies. Indeed, other researchers have recently suggested the relevance of the rs3757441 *EZH2* SNP in hepatocellular carcinoma: Yu et al. reported that patients who carried at least one C allele have a higher lymph-node-metastasis risk than did patients carrying the wild-type allele [16], thus confirming the potential functional relevance of this SNP in cancer cells. Secondly, a standardized score to evaluate EZH2 (and H3K27me3) positivity by immunohistochemistry is lacking: future studies should thus clarify which is the preferred method to assess this parameter (i.e. the percentage of positive cells, the intensity of staining or a combination of both, as for the SI proposed by other authors [13]).

## Conclusions

To conclude, we characterized the *EZH2* rs3757441 SNP, showing that the C/C variant is associated with higher EZH2 expression and activity in the neoplastic tissue. This SNP genotype has been linked with worse prognosis among mCRC patients treated with chemotherapy with or without an anti-vascular endothelial growth factor antibody: our data confirm in primary tumors that the C allele is linked with increased EZH2 expression and activity, thus providing additional evidence for a functional relevance of this SNP in CRC biology. As anti-EZH2 drugs are under development and early clinical evaluation [17], this SNP may serve as a potential predictive biomarker for such agents in CRC patients.

## Additional file

Additional file 1: Table S1: Correlation of EZH2 rs3757441 variants with clinical and molecular characteristics. Abbreviations: %, percentage; *n*, number; *P*, *P*-value. **Table S2:** EZH2 expression according to tumour characteristics. Abbreviations: %, percentage; *n*, number; *P*, *P*-value; SD, standard deviation; SI, Staining Index. **Table S3:** H3K27me3 expression according to tumour characteristics. Abbreviations: %, percentage; *n*, number; *P*, *P*-value; SD, standard deviation. (PDF 98 kb)

## Abbreviations

CRC: colorectal cancer
H3K27me3: histone H3 trimethylation on lysine 27
mCRC: metastatic colorectal cancer
PcGs: Polycomb Group genes
PCR: polymerase chain reaction
PRCs: Polycomb Repressive Complexes
SNPs: single-nucleotide polymorphisms
