# Supplementary material for: Molecular and pathological characterization of the EZH2 rs3757441 single nucleotide polymorphism in colorectal cancer
Source: BMC Cancer. 2015 Nov 9;15:874. doi: 10.1186/s12885-015-1889-2 (PMC4640238; doi:10.1186/s12885-015-1889-2)
Supplement: Additional file 1: Table S1: — Correlation of EZH2 rs3757441 variants with clinical and molecular characteristics. Abbreviations: %, percentage; n, number; P, P-value. Table S2: EZH2 expression according to tumour characteristics. Abbreviations: %, percentage; n, number; P, P-value; SD, standard deviation; SI, Staining Index. Table S3: H3K27me3 expression according to tumour characteristics. Abbreviations: %, percentage; n, number; P, P-value; SD, standard deviation. (PDF 98 kb) [file 12885_2015_1889_MOESM1_ESM.pdf]

Additional Table 1: Correlation of *EZH2* rs3757441 variants with clinical and molecular characteristics

	EZH2 genotype			
	**C/C (*n*=5)**	**C/T (*n*=51)**	**T/T (*n*=63)**	***P***
Tumor stage				
I	0 (0%)	1 (2%)	4 (6%)	
II	3 (60%)	19 (37%)	19 (30%)	
III	2 (40%)	21 (41%)	29 (47%)	
IV	0 (0%)	10 (20%)	11 (17%)	0.667
Tumor grade				
2	0 (0%)	32 (63%)	39 (62%)	
3-4	5 (100%)	19 (37%)	24 (38%)	0.021
Tumor site				
right colon	3 (60%)	19 (37%)	30 (48%)	
left colon	2 (40%)	17 (33%)	26 (41%)	
rectum	0 (0%)	15 (30%)	7 (11%)	0.109
Mucinous histology				
yes	0 (0%)	14 (27%)	23 (37%)	
no	5 (100%)	37 (73)	40 (63%)	0.180

KRAS status				
wild-type	3 (60%)	30 (59%)	34 (54%)	
mutant	2 (40%)	21 (41%)	29 (46%)	0.897
BRAF status				
wild-type	5 (100%)	50 (98%)	53 (84%)	
mutant	0 (0%)	1 (2%)	10 (16%)	0.030

Abbreviations: %, percentage; *n*, number; *P*, *P*-value.

Additional Table 2: EZH2 expression according to tumour characteristics

	3+ staining intensity		% positive cells		SI 4-9	
**Variable (*n*)**	***n* (%)**	***P***	**mean (SD)**	***P***	***n* (%)**	***P***
Tumor stage						
I (5)	1 (20%)		48% (25.88)		5 (100%)	
II (41)	22 (54%)		47.93% (28.92)		30 (73%)	
III (52)	22 (42%)		44.9% (30.25)		35 (67%)	
IV (21)	10 (48%)	0.558	45.71% (28.39)	0.966	17 (81%)	0.336
Tumor grade						
2 (76)	39 (51%)		47.3% (27.9)		59 (78%)	
3-4 (43)	16 (37%)	0.181	45.3% (30.4)	0.722	28 (65%)	0.196
Tumor site						
right colon (52)	21 (40%)		49.8% (28.9)		38 (73%)	
left colon (45)	25 (56%)		42.8% (29.2)		32 (71%)	
rectum (22)	9 (41%)	0.271	46.8% (27.8)	0.489	17 (77%)	0.867
Mucinous histology						
Yes (37)	17 (46%)		52.2% (30.1)		28 (76%)	
No (82)	38 (46%)	1.000	44.1% (27.9)	0.157	59 (72%)	0.824

KRAS status						
wild-type (67)	30 (45%)		45.3% (30.2)		49 (73%)	
mutant (52)	25 (48%)	0.853	48.2% (26.9)	0.600	38 (73%)	1.000
BRAF status						
wild-type (108)	50 (46%)		46.3% (28.8)		78 (72%)	
mutant (11)	5 (45%)	1.000	49.1% (29.8)	0.764	9 (82%)	0.725

Abbreviations: %, percentage; *n*, number; *P*, *P*-value; SD, standard deviation; SI, Staining Index.

Additional Table 3: H3K27me3 expression according to tumour characteristics

	3+ staining intensity		% positive cells	
**Variable (*n*)**	***n* (%)**	***P***	**mean (SD)**	***P***
Tumor stage				
I (5)	1 (20%)		60.0% (28.28)	
II (41)	14 (34%)		60.2% (30.78)	
III (52)	24 (46%)		64.8% (29.14)	
IV (21)	9 (43%)	0.566	71.4% (26.13)	0.541
Tumor grade				
2 (76)	36 (47%)		67.9% (28.02)	
3-4 (43)	12 (28%)	0.052	57.7% (30.15)	0.065
Tumor site				
right colon (52)	17 (33%)		65.8% (27.18)	
left colon (45)	25 (55%)		67.8% (29.15)	
rectum (22)	6 (27%)	0.028	53.2% (31.98)	0.136

Mucinous histology				
Yes (37)	11 (30%)		62.97% (27.88)	
No (82)	37 (45%)	0.157	64.79% (29.76)	0.758
KRAS status				
wild-type (67)	30 (45%)		64.03% (30.35)	
mutant (52)	18 (35%)	0.346	64.42% (27.68)	0.942
BRAF status				
wild-type (108)	42 (39%)		62.96% (30.09)	
mutant (11)	6 (55%)	0.347	76.36% (11.20)	0.146

Abbreviations: %, percentage; *n*, number; *P*, *P*-value; SD, standard deviation.
